# Long way up: rethink diseases in light of phase separation and phase transition

**DOI:** 10.1093/procel/pwad057

**Published:** 2023-12-09

**Authors:** Mingrui Ding, Weifan Xu, Gaofeng Pei, Pilong Li

**Affiliations:** State Key Laboratory of Membrane Biology & Frontier Research Center for Biological Structure, School of Life Sciences, Tsinghua University, Beijing 100084, China; Tsinghua-Peking Center for Life Sciences, Beijing 100084, China; NuPhase Therapeutics, Beijing 100083, China; State Key Laboratory of Membrane Biology & Frontier Research Center for Biological Structure, School of Life Sciences, Tsinghua University, Beijing 100084, China; Tsinghua-Peking Center for Life Sciences, Beijing 100084, China; NuPhase Therapeutics, Beijing 100083, China; State Key Laboratory of Membrane Biology & Frontier Research Center for Biological Structure, School of Life Sciences, Tsinghua University, Beijing 100084, China; Tsinghua-Peking Center for Life Sciences, Beijing 100084, China; State Key Laboratory of Membrane Biology & Frontier Research Center for Biological Structure, School of Life Sciences, Tsinghua University, Beijing 100084, China; Tsinghua-Peking Center for Life Sciences, Beijing 100084, China

**Keywords:** multivalency, compartments, aberrant phase separation, therapeutics, gain or loss of phase separation, diseases

## Abstract

Biomolecular condensation, driven by multivalency, serves as a fundamental mechanism within cells, facilitating the formation of distinct compartments, including membraneless organelles that play essential roles in various cellular processes. Perturbations in the delicate equilibrium of condensation, whether resulting in gain or loss of phase separation, have robustly been associated with cellular dysfunction and physiological disorders. As ongoing research endeavors wholeheartedly embrace this newly acknowledged principle, a transformative shift is occurring in our comprehension of disease. Consequently, significant strides have been made in unraveling the profound relevance and potential causal connections between abnormal phase separation and various diseases. This comprehensive review presents compelling recent evidence that highlight the intricate associations between aberrant phase separation and neurodegenerative diseases, cancers, and infectious diseases. Additionally, we provide a succinct summary of current efforts and propose innovative solutions for the development of potential therapeutics to combat the pathological consequences attributed to aberrant phase separation.

## Introduction

Eukaryotes coordinate the cooperative functioning of cellular components through compartmentalization, achieved via classical membrane-bound organelles, and membrane-less organelles (MLOs), also known as biomolecular condensates (Banani et al., 2017; Lyon et al., 2021). MLOs are formed, in part, through biomacromolecular liquid–liquid phase separation (LLPS). Numerous studies have demonstrated the remarkable liquid properties of MLOs (Hyman et al., 2014; Shin and Brangwynne, 2017). These MLOs exhibit efficient biological reaction kinetics, rapid assembly and disassembly, functional sorting capacity, and unique physical morphology (Hyman et al., 2014). Biomolecular condensates are found within the cytoplasm, nucleus, and attached to membranes (Lyon et al., 2021). Multivalent interactions between folded domains and/or within certain types of intrinsically disordered regions (IDRs) underlie LLPS (Li et al., 2012; Wang et al., 2018b). Leveraging this principle, cells autonomously optimize intracellular biochemical activities. These intriguing discoveries prompt a compelling reevaluation of cell biology (Boeynaems et al., 2018).

Now that a wide range of biomolecular condensates has been explored, the field is poised to uncover the connections between phase separation and cellular function. Recently, a number of studies have sought to elucidate the relationship between aberrant phase separation and its association with neurodegenerative diseases, cancer, and infectious diseases (Aguzzi and Altmeyer, 2016; Alberti and Dormann, 2019; Peng et al., 2020a; Shin and Brangwynne, 2017; Taniue and Akimitsu, 2021; Zbinden et al., 2020). Studies are emerging to uncover the consequences of disease mutations on phase separation behaviors. These consequences encompass the loss, gain and translocation of phase separation, alterations in the components within the condensates, transitions from liquid to gel or solid condensates, and other alterations in the physical properties of condensates (Alberti and Dormann, 2019; Shin and Brangwynne, 2017; Taniue and Akimitsu, 2021; Zbinden et al., 2020). These studies encouraged us to re-think diseases through the lens of aberrant phase separation, leading to a deep understanding of diseases and eventually novel therapies.

**Figure 1.**
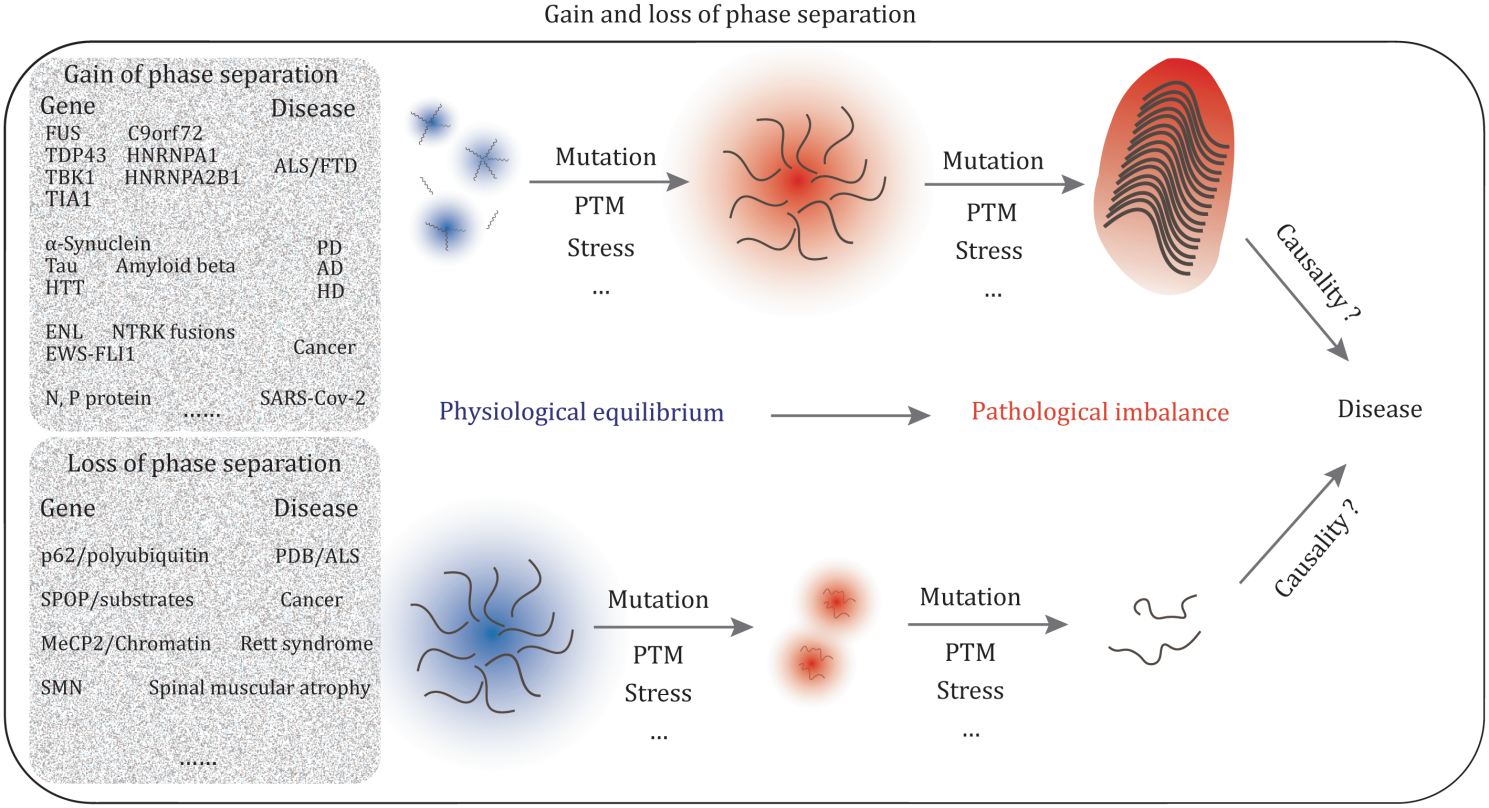
Gain and loss of phase separation and their potential links with diseases. Top: genomic variants, post translation modifications (PTM), stress, or other abnormal conditions coupled with acquiring further phase separation ability (LLPS enhancement, liquid to gel/glass/solid transition), termed gain of phase separation (GoPS). Bottom: on the other side, original physiological LLPS partially or totally lose phase separation, termed loss of phase separation (LoPS). Nevertheless, the causality between gain and loss of phase separation with disease is still elusive.

Mutations occur constantly in genomes across all organisms, including humans. While most variants have neutral effects, some can lead to consequences in the host organisms, particularly causing human diseases. Disease-causing mutations are typically classified as loss-of-function or gain-of-function variants (Li et al., 2019; Lugo-Martinez et al., 2016). Similarly, it is advantageous to categorize aberrant phase separation resulting from disease mutations as loss of phase separation (LoPS) and gain of phase separation (GoPS) (Fig. 1). In brief, we define defective condensates when they exhibit limited phase-separating ability or even disassembly, which falls under LoPS. In contrast, GoPS represents those with newly acquired phase separation or a transitioned state (gel or glass-like) (Fig. 1).

While existing evidence has shed light on a variety of pathological consequences that arise from both the LoPS and GoPS, the exact causality remains elusive. It is important to acknowledge the absence of absolute patterns when considering the two facets of phase separation in diseases, as losses and gains can even occur in combination. In summary, aberrant phase separation, as represented by LoPS and GoPS, is increasingly recognized for its connection to various diseases, including neurodegenerative conditions, cancer, and infectious diseases. Therefore, this review aims to provide a fresh perspective on the intricate relationship between aberrant phase separation and diseases, while also speculating on potential therapeutic opportunities for addressing these diseases.

## Aberrant phase separation and neurodegeneration

Neurodegenerative diseases pose a growing burden on healthcare systems worldwide. Prominent neurodegenerative diseases include Alzheimer’s disease (AD), amyotrophic lateral sclerosis (ALS), Friedreich’s ataxia (FA), frontotemporal dementia (FTD), Huntington’s disease (HD), Parkinson’s disease (PD) (Abramzon et al., 2020; Gitler et al., 2017; Peng et al., 2020a). Over the past few decades, a wealth of evidence suggests that protein aggregation is a hallmark feature of some of these diseases (Floeter and Gendron, 2018; Gitler et al., 2017; Peng et al., 2020a). Several proteins implicated in neurodegenerative diseases are known to form distinct biomolecular condensates. For instance, TDP-43, FUS, Tau, hnRNPA1, hnRNPA2B1, poly-glutamine (polyQ) containing protein, and TIA1 are components of various types of ribonucleoprotein (RNP) condensates that regulate the fate of RNA molecules during processes such as splicing, nuclear export, cytoplasmic trafficking, translation, and degradation (Ash et al., 2021; Grese et al., 2021; Hallegger et al., 2021; Levone et al., 2021; Maharana et al., 2018; Ritsch et al., 2022).

Mechanistically, aberrant phase separation can lead to both GoPS, promoting the transition from liquid to solid and oligomer to fibrils, and LoPS, which are associated with cellular pathological changes. These irreversible biophysical properties have been linked to various diseases affecting cellular phenotypes. Since the phase separation paradigm has deepened our understanding of neurological disorders, this review aims to summarize these lines of study and synthesize the correlations between aberrant phase separation and neurodegeneration.

### Aberrant phase separation in ALS and FTD

The most prevalent neurodegenerative diseases associated with aberrant phase separation are ALS and FTD. ALS primarily affects motor neurons, while FTD predominantly affects cerebral neurons (Alberti and Dormann, 2019). Accumulating evidence suggests that ALS and FTD patients exhibit significant clinical and neuropathological overlap, especially in changes related to eating behavior and metabolism (Ahmed et al., 2016; Weishaupt et al., 2016). Approximately 10%–15% of patients diagnosed with FTD also display symptoms of ALS (Ferrari et al., 2011). In FTD, motor neuron dysfunction, which does not meet the criteria for an ALS diagnosis, is observed in approximately 25% of cases (Abramzon et al., 2020). Moreover, these diseases share overlapping genetic abnormalities, involving genes such as SQSTM1 (p62), TDP43, FUS, TBK1, TIA1, CHCHD10, hnRNPA1, hnRNPA2B1, MATR3, and C9orf72, which have been implicated in autophagy and RNA metabolism (Abramzon et al., 2020; Floeter and Gendron, 2018).

**Figure 2.**
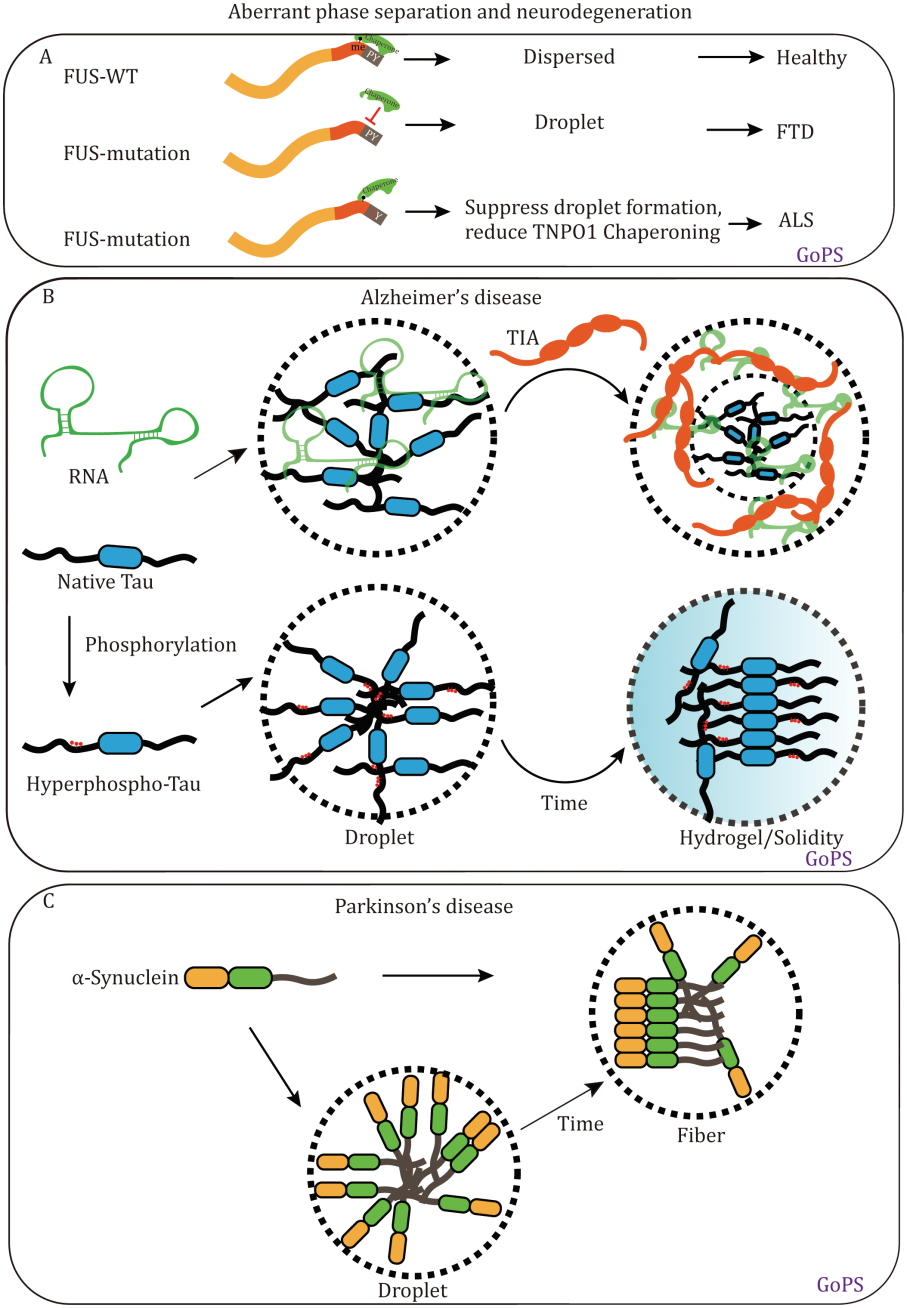
Aberrant phase separation with neurodegenerative diseases. (A) Liquid–liquid phase separation of FUS in healthy and gain of phase separation in FTD, ALS diseases. (B) RNA and PTM promote Tau protein from liquid droplet to hydrogel transition in Alzheimer disease. (C) Continuous accumulation of α-Synuclein causes the formation of fiber in Parkinson’s disease.

Building upon the previous discussion, it is worth noting that a wealth of evidence supports the involvement of aberrant phase separation of RNA-binding proteins in ALS and FTD. Mutations associated with these diseases, including FUS, TDP43, hnRNPA1, hnRNPA2B1, and TIA1, contribute to the accumulation of associated proteins within stress granules (SGs) and the acquisition of phase separation properties (Alami et al., 2014; Floeter and Gendron, 2018; Kim et al., 2013; Mackenzie et al., 2017). In patients, the aggregation of FUS and TDP43 also leads to the selective recruitment of PABPC1 and TIA1, thereby disrupting RNA metabolism, the dynamics of RNP granules, and the homeostasis of RNA-binding proteins (Mackenzie et al., 2017). Mutations in FUS, hnRNPA1, and TDP43 associated with ALS and FTD accelerate the aberrant transition from liquid to solid state, resulting in the loss of function of diverse RNP granules and eventually leading to pathological processes (Alberti and Dormann, 2019; Patel et al., 2015). In addition, the regulation of internal post-translational modifications (PTMs) also contributes to pathological phase separation (Fig. 2A). In patients, the arginine residues within the RGG/RG-rich IDRs of FUS remain unmethylated, which promotes phase separation and reduces the dynamic of FUS condensates both in cellular and *in vitro* (Fig. 2A). Besides that, TDP43 is hyperphosphorylated in ALS and FTD patients, which contributes to an increased velocity of protein aggregation (Suarez-Calvet et al., 2016). RNA, being a crucial modulator for RNA-binding protein phase separation, has recently sparked a contentious debate regarding its role in regulating protein aggregation. Several studies propose that RNA binding mediates protein aggregation and neurotoxicity (Flores et al., 2019; Voigt et al., 2010). Conversely, others suggest the occurrence of aggregation through an RNA-independent pathway (Mann et al., 2019). Moving beyond the debate of whether RNA plays distinct roles in neurodegenerative disease, it is crucial to recognize the significance of RNA metabolism, which includes processes such as RNA editing, degradation, and exportation. Interestingly, RNA modifications also play an important role in the interaction of RNA and protein. For example, RNA methylation serves as a trigger for phase separation (Alriquet et al., 2021; Gao et al., 2019; Liu et al., 2020; Ries et al., 2019; Wang et al., 2020). So, alterations of RNA metabolism also can lead to aberrant phase separation of RNA binding protein during the pathological processes of ALS and FTD.

Recent studies have shed light on the significant influence of ATP and small molecules on phase separation. Lower ATP concentrations promote the organization of FUS into droplets, whereas higher ATP concentrations disrupt LLPS. This behavior can be attributed to the multivalent interaction of ATP at low concentrations and the exposure of hydrophobic characteristics at high concentrations (Kang et al., 2018). Similarly, the ALS-linked mutation of TDP-43 hinders its interaction with ATP (Dang and Song, 2020). Overall, the aggregation or aberrant phase separation mentioned above represents major hallmarks of GoPS in ALS and FTD.

### Aberrant phase separation in AD

The microtubule-associated protein Tau was first purified in 1975 and its pathological accumulation is considered a hallmark of AD (Sengoku, 2020). Accumulating evidence found that full-length Tau undergoes LLPS to form droplets in physiological conditions (Boyko et al., 2019; Kosik and Han, 2019; Savastano et al., 2021). Notably, Tau exhibits distinct phase separation properties in healthy neurons compared to those in AD neurons. The liquid compartment of Tau efficiently increases tubulin concentration and facilitates the nucleation of microtubules (Ambadipudi et al., 2017). Disease mutation or abnormal post-transcription modifications in Tau promotes the transition from liquid phase to solid phase, especially through the hyperphosphorylation of Tau in AD and FTD patients, which extends its phase separation ability (Savastano et al., 2021) (Fig. 2B). We refer to this type of aberrant phase separation as GoPS (Fig. 2B). Moreover, since Tau is required for the normal interaction of RNA binding protein in brain tissue, aberrant phase separation of Tau leads to accumulating toxicity and dysfunctional RNA metabolism (Ambadipudi et al., 2017; Savastano et al., 2021) (Fig. 2B).

### Aberrant phase separation in HD

HD is a classical genetic neurodegenerative disease caused by the expansion of CAG triplet repeats in the first exon of Huntingtin (HTT) gene (Jimenez-Sanchez et al., 2017). Aggregation of mutant HTT (mHTT), both alone and in conjunction with other proteins, is a hallmark of HD; however, the mechanism underlying mHTT aggregation remains puzzling (DiFiglia et al., 1997; Jimenez-Sanchez et al., 2017). Numbers of recent studies indicate that mHTT exhibits phase separation properties both in cellular and *in vitro* (Aktar et al., 2019; Posey et al., 2018). Base on solubility analysis, right-angle static light scattering, and transmission electron microscopy, it has been observed that the N-terminal of HTT undergoes three distinct phases *in vitro*, including the M phase (monomer and oligomer), S phase (bigger soluble aggregates around 25 nm diameter), and F phase (fibrillar aggregates (Posey et al., 2018)). Moreover, mHTT exon1 product (mHTTEX1) in yeast has been also observed to undergo a liquid-to-solid phase transition (Aktar et al., 2019). By constructing different lengths of mHTTEX1-polyQ, the results suggest that the expanded polyQ is extremely vital for mHTT condensates behavior and kinetics of solid transition (Aktar et al., 2019). It is critical to answer the pathological relevance between the complicated gain of aberrant mHTT phase separation and HD. Extensive discussion and deep thinking are needed to unravel whether all HD patients’ cell performs universal mechanism, especially in diverse types of neurons, and how internal PTMs and the external environment disturb functional soluble phase state, eventually leading to irreversible toxic aggregation (Yang and Yang, 2020). Overall, antagonizing or reversing the pathological aberrant mHTT phase separation holds potential as a next-generation therapeutics for HD.

### Aberrant phase separation in PD

PD is a prevalent neurodegenerative disorder that affects 1%–3% of aging population (Wyss-Coray, 2016). Clinical features of PD comprise resting tremor, rigidity, postural instability, and bradykinesia. α-Synuclein (α-Syn) aggregation and amyloid accumulation are highly linked with PD (Jankovic, 2008; Lotankar et al., 2017). However, the initiating events of aggregation remain elusive and aberrant phase transition among distinct phases has emerged as a potential novel perspective to extend our understanding of PD. Recently, N-terminal and hydrophobic non-amyloid-β component (NAC) domain of α-Syn have been reported as major drivers of LLPS (Ray et al., 2020) (Fig. 2C). Interestingly, α-Syn droplets perform liquid-to-solid phase transition after 20 days *in vitro* and this GoPS property holds crucial pathological relevance (Ray et al., 2020) (Fig. 2C). PD-associated aggregation factors promote α-Syn phase separation. Substantial PTMs of α-Syn including phosphorylation, SUMOylation, nitration, and O-GlcNAcylation significantly influence its solubility and toxicity (Ray et al., 2020). Specific regulation signatures serve as vital switches to alter phase separation behavior and material property, eventually initiating pathological processes. While numerous *in vitro* studies suggest a connection between the abnormal phase separation of α-Syn and PD, a more comprehensive understanding in *in vivo* models still requires further exploration.

### Autophage and neurodegeneration

Recent accumulating evidence suggests that the autophage system has a general ability to undergo phase separation, whereas disease-related mutations result in a LoPS. Given that aberrant phase separation or protein aggregation is a novel hallmark of neurodegeneration, autophage serves as a complex security mechanism to heal improperly folded proteins. Destruction of the autophage system leads to various severe effects on pathological neurodegeneration (Dubnikov et al., 2017). Misfolded proteins or aggregates must be degraded following these fundamental principles. Remarkably, polyubiquitin chain-induced p62 phase separation drives autophagic cargo segregation (Danieli and Martens, 2018; Sanchez-Martin and Komatsu, 2018; Sun et al., 2018).

**Figure 3.**
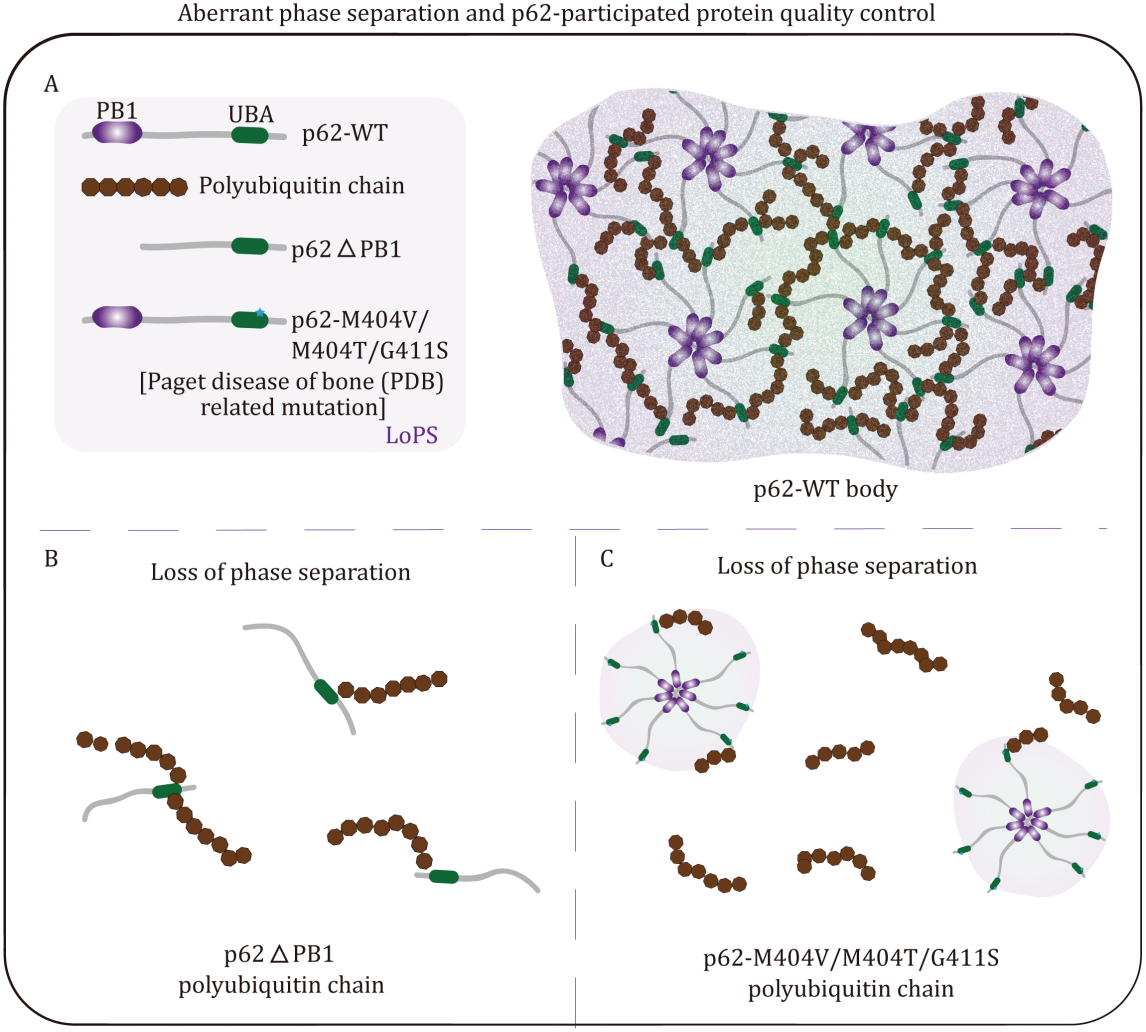
Loss of phase separation in p62-participated protein quality control. (A) Left: schematic of p62-WT, polyubiquitin chain, p62ΔPB1 and Paget’s disease of bone (PDB) mutation of p62-M404V/M404T/G411S. PB1: Phox 1 and Bem1p domain for oligomerization. UBA: ubiquitin associated domain for ubiquitin binding. Right: multivalent assembly of p62 with polyubiquitin chain contribute to p62 liquid–liquid phase separation. (B) p62ΔPB1 loses phase separation with polyubiquitin chain due to valency deficiency. (C) PDB related mutations of p62 compromise physical interaction with polyubiquitin chain and hence loss of phase separation.

p62, a major component for diverse cellular protein degradation systems, has been implicated in both Paget’s disease of bone (PDB) and ALS. The basic function of p62 in autophagy is well known, as it acts as a connector between ubiquitin-modified proteins and autophagosomes for degradation of the former. Mechanistically, p62 undergoes phase separation with polyubiquitin and is regulated by post-translational modification (Fig. 3A). Importantly, mutations in p62 associated with PDB and the p62-ΔPB1 variant dramatically disrupt the physical interaction with ubiquitin chain and the oligomerization of p62, resulting in the LoPS in cellular and *in vitro* (Fig. 3B and 3C). Importantly, the valency of p62 body is critical for the multivalent assembly of LLPS. Along this way, the potential intervals could be a reversion of monomer to oligomer wherein LoPS of p62 disease mutations. In addition, the proteasome represents another collective protein quality control pathway. Proteasome-containing nuclear foci, comprising ubiquitylated proteins and many proteasome-binding components, form liquid condensates through multivalent interaction (Cohen-Kaplan et al., 2020; Yasuda et al., 2020). Hyperosmotic stress induces the formation of liquid foci and promotes phase separation. Mechanically, polyubiquitin chain and RAD23B provide essential roles in triggering condensate formation by providing the multivalency for phase separation. Moreover, acute hyperosmotic stress may risk much aggregation accumulation leading to the gain of phase transition from liquid-to-solid. Thus, timely clearance of toxic aggregation is extremely vital for protein homeostasis and disease progression. Increasing evidence reveals the profound functional consequences and physiological relevance of degradation condensates. Considering these aspects could be critical for the development of next-generation therapeutic interventions for neurodegenerative diseases.

## Aberrant phase separation and cancer

Cancer, the second leading cause of death around the world, claims the lives of more than eight million people every year (Gerstung et al., 2020). The complicated genetic mutations associated with cancer result in massive dysfunction across the molecular scale to organism level (Gerstung et al., 2020; Li et al., 2020; Rodriguez-Martin et al., 2020). Despite the genetic heterogeneity of cancer, it exhibits common features such as abnormal cell proliferation, invasion, metastasis, and metabolism (Hanahan and Weinberg, 2011). However, there are still significant limitations in cancer therapeutics. The concept of phase separation could provide a novel perspective to understand the complex characteristics of cancer. A number of studies have attempted to establish the relationship between phase separation and cancer; however, only a fraction of these studies highlight central valuable information and provide solid evidence. So, we have picked out some fundamental works to discuss, focusing on both the loss of function and the gain of function mechanisms.

**Figure 4.**
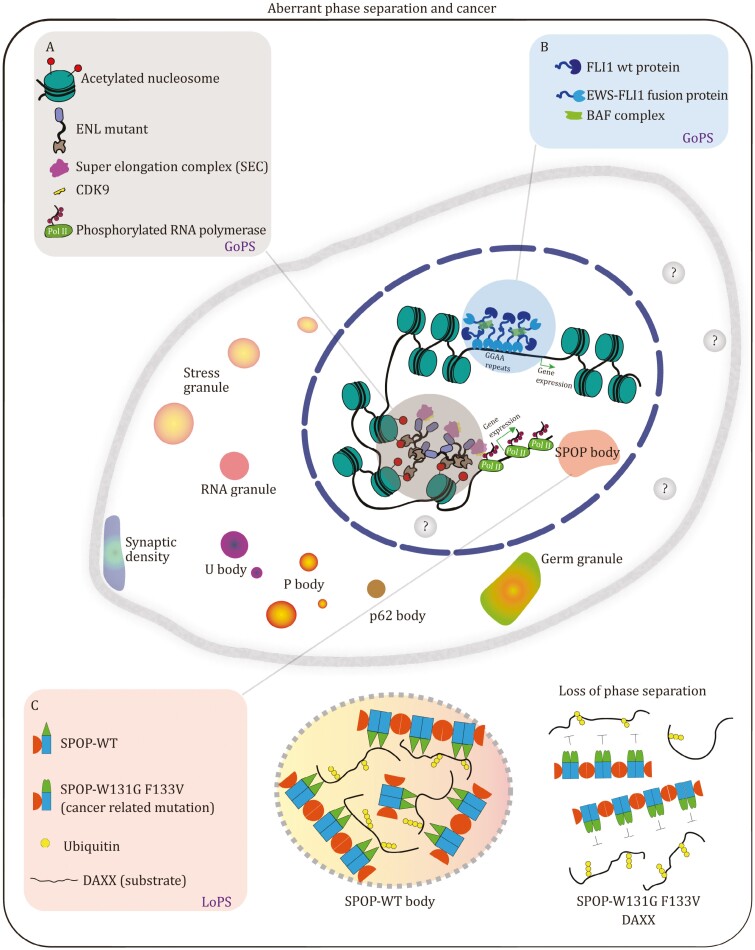
Aberrant phase separation and cancer. (A) Cancer-related mutants of ENL form condensates within nucleus and promote recruitment and transcription of elongation. (B) EWS-FLI1 fusion protein in diseases acquires phase separation ability and disturbs downstream gene regulation. (C) Cancer related mutations of SPOP compromise its phase separation with substrates.

One key progress has been made regarding gain-of-function mutations in the eleven-nineteen-leukemia protein (ENL) and its association with human Wilms tumor (Guo et al., 2020; Wan et al., 2020). ENL, acting as a reader of histone acetylation, plays an important role in the oncogenic state of leukemia (Guo et al., 2020; Wan et al., 2020). Compared with wide-type ENL, tumor-associated mutations result in the formation of micrometer-sized condensates, which enhance the recruitment and activity of transcription elongation (Guo et al., 2020; Wan et al., 2020). The tumor-related mutations enhance self-association and involve extra dynamic multivalent interactions, which leads to gain-of-function condensates (Fig. 4A).

Another example of tumor arises due to chromosome translocations, leading to various fusion gene products (Delattre et al., 1992; Tan and Manley, 2009) (Fig. 4B). The common features of those new proteins typically exhibit the fusion of intrinsic disorder region with DNA or chromatin binding domain (Kovar, 2011). This kind of specific sequence characteristics results in phase separation. Classic examples include the N-terminal IDR of EWSR1 and the DNA binding domain of FLI-1 in Ewing’s sarcoma and the N-terminal IDR of FUS and transcription factor CHOP in liposarcoma (Delattre et al., 1992; Shorter, 2017) (Fig. 4B). EWS-FLI1 and FUS-CHOP fusion proteins frequently GoPS within the nucleus and lead to dysfunctional downstream gene regulation (Tan and Manley, 2009) (Fig. 4B). Recently, an increasing number of fusion proteins with GoPS are performing diverse functions in various subcellular locations (Tripathi et al., 2023).

Tumor suppressor speckle-type POZ protein (SPOP) is cullin 3 (CUL3)-based E3 ligase substrate-binding subunit and frequently mutated in solid cancers (Bouchard et al., 2018; Seton-Rogers, 2018) (Fig. 4C). Tumor-related mutations of SPOP are commonly found in the substrate-binding meprin and TRAF homology domain. SPOP substrates typically comprise death-domain-associated protein (DAXX), androgen receptor, epigenetic modifiers, and hormone signaling effectors (Fig. 4C). Mittag and coworkers discovered that the phase separation of SPOP and substrate depend on multivalent interaction, which is disrupted by cancer mutations (Bouchard et al., 2018) (Fig. 4C). To sum up, LoPS of SPOP provides a novel version to understand genetic variants.

Collectively, increasing studies suggest that phase separation supplies a mechanism to rethink how the fundamental transcription process works by rule and line. We also need to develop specific strategies to rescue disease-causing aberrant phase separation.

## Aberrant phase separation and infectious diseases

Over the past decade, substantial experimental evidence has been amassed, indicating the significant role of biomolecular condensates in the context of infectious diseases. These biological condensates play a pivotal role in shaping the spatio-temporal organization of cellular components. Notably, pathogens exploit these gain-of-condensates to enhance their infectivity, while these same structures are harnessed by host defense mechanisms for pathogen detection and neutralization. In this review, we comprehensively sum up the existing data pertaining to the LLPS specifically triggered by biomolecules of both viral and host origins, shedding light on their functional implications.

### Virus infection

LLPS induced by viral proteins has been implicated in a diverse spectrum of pivotal steps and regulatory processes throughout viral life cycles. Given that viral proteins often exhibit enrichment in IDRs and possess multivalency, their propensity for LLPS is unsurprising, as demonstrated by recent investigations providing experimental validation of this phenomenon and elucidating its functional ramifications. We differentiate LLPS events associated with viral replication, the assembly of viral components, and the intracellular trafficking of viral materials, from instances where LLPS mediates the disruption of host cell functions by viruses.

**Figure 5.**
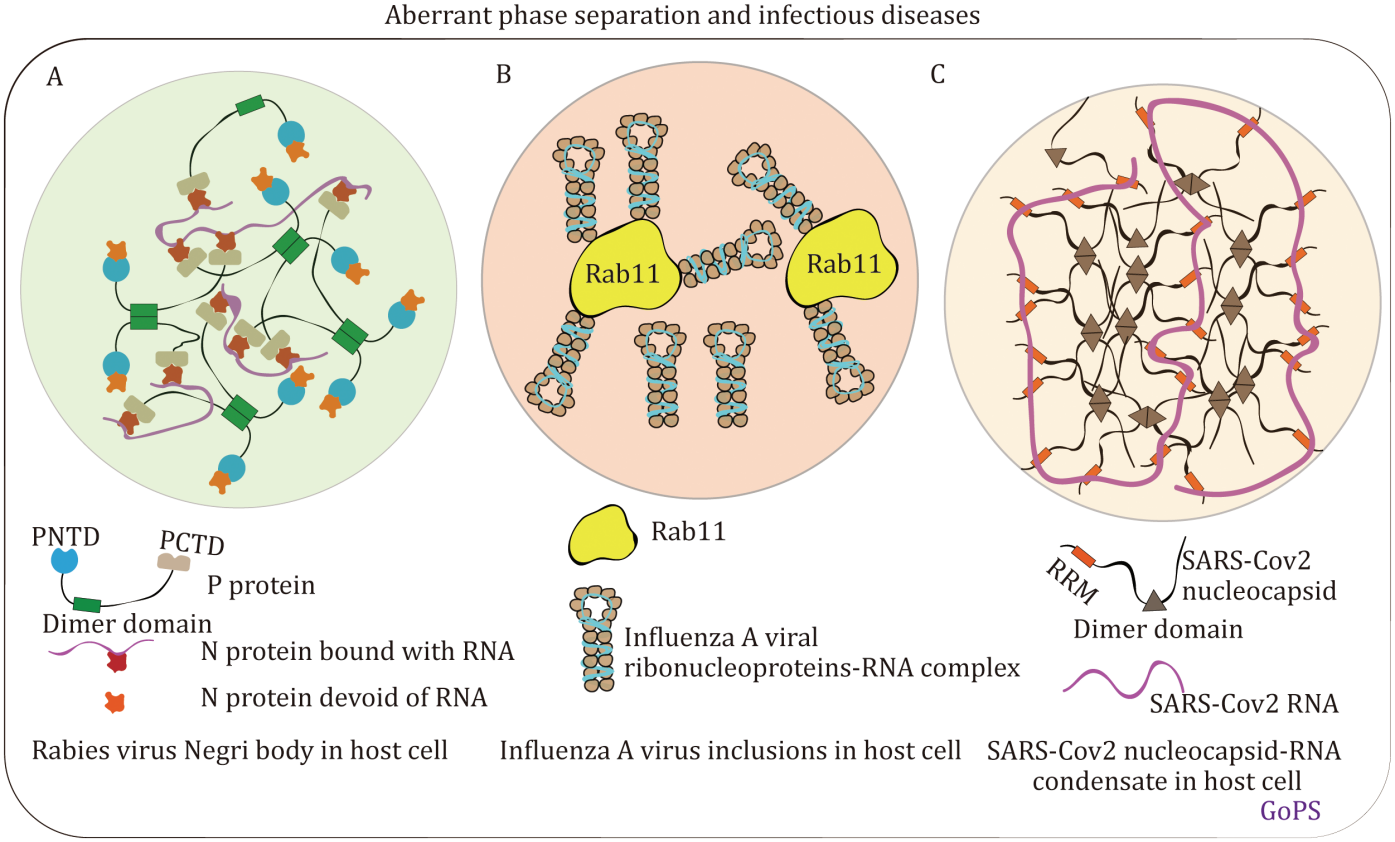
Representative examples for disturbed phase separation and condensate formation in virus infection. (A) Rabies virus N and P proteins assemble into Negri bodies as liquid-like viral factories. (B) Influenza A virus ribonucleoproteins form liquid inclusion at endoplasmic reticulum exit sites. (C) SARS CoV-2 nucleocapsid protein forms condensates with viral genomic RNA.

A functionally significant manifestation of LLPS orchestrated by viral proteins is the formation of condensate-like structures commonly referred to as “replication compartments” (RCs) or “inclusion bodies” (IBs) (Nikolic et al., 2017; Guseva et al., 2020; Risso-Ballester et al., 2021). RCs serve as specialized sites where viral replication and assembly processes occur, facilitating the concentration of specific viral and cellular proteins, as well as nucleic acids. These compartments act as platforms that optimize viral replication, primarily through the selective inclusion or exclusion of components and shielding against host immune defenses. Viral factories can exhibit either membrane-delimited structures or exist in a non-membrane-bound form. The first documented instance of LLPS by viral proteins *in vivo* pertains to the rabies virus (RABV) (Nikolic et al., 2017). A distinctive feature of RABV infection is the presence of cytoplasmic inclusions known as Negri bodies (NBs) (Fig. 5A). In this case, the use of immunofluorescence, coupled with electron microscopy, facilitated the identification of cytoplasmic inclusions. These inclusions lacked a surrounding membrane and were categorized based on their sizes. Subsequently, live cell imaging provided insights into the dynamic behavior of these non-membrane-bound structures known as NBs. It revealed their capacity to fuse with one another and showcased their rapid internal diffusion, as assessed through fluorescence recovery after photobleaching (FRAP) analysis (Nikolic et al., 2017). Furthermore, the emergence of a simplified NB-like system when only the RABV N and P proteins are expressed underscores the pivotal role of these viral proteins in driving LLPS. Reevaluating the existing body of literature, it becomes evident that the formation of RCs via LLPS, catalyzed by viral proteins, is a recurring characteristic shared by a majority of RNA viruses. This phenomenon was promptly demonstrated in the case of the vesicular stomatitis virus (Heinrich et al., 2018), Measles virus (MeV) (Zhou et al., 2019), and respiratory syncytial virus (Galloux et al., 2020). Notably, the maturation of IBs in MeV exhibits a more gel-like property (Zhou et al., 2019), as inferred from FRAP analysis, suggesting a possible regulatory mechanism controlling the dynamics of compartments to optimize viral infection.

What is particularly fascinating is that viral condensates, induced by LLPS, extend their influence beyond the sites of viral RNA synthesis. They are intricately linked to the processes of virion assembly and trafficking. Consider the case of the influenza A virus (IAV), as depicted in Fig. 5B. Here, viral inclusions positioned in close proximity to endoplasmic reticulum (ER) exit sites serve to segregate viral ribonucleoproteins (vRNPs) from the surrounding cytosol. This segregation creates a dynamic, liquid-like environment that facilitates specific RNA–RNA interactions. As a result, it enables precise spatiotemporal control over the assembly process of the eight-segmented RNA genome. This phenomenon represents a remarkable illustration of the multifaceted roles of LLPS in viral biology (Alenquer et al., 2019). Besides, viral tegument protein, i.e., UL11 from Herpes simplex virus 1 (HSV-1), has also been shown to be endowed with a high degree of structural disorder and multivalent interaction properties for undergoing LLPS *in vitro* (Metrick et al., 2020). Considering its interaction with multiple viral partners and RNA (Chadha et al., 2012; Han et al., 2012; Klupp et al., 2005; Yeh, 2009), tegument protein UL11 is likely to form biomolecular condensates to exert distinct roles in tegument assembly and final stages of virus maturation. Another example refers to the human immunodeficiency virus 1 (HIV-1) nucleocapsid protein (NC) (Rice et al., 1995), a cleavage product generated from pr55^Gag^ controlling crucial steps of retroviral replication (Abd El-Wahab et al., 2014; Bernacchi et al., 2017). As a Zn finger protein, NC undergoes Zn^2+^-dependent LLPS, and Zn^2+^ chelation impairs the colocalization of NC and viral RNA, as well as their trafficking between the cytoplasm and the nucleus (Monette et al., 2020).

As a hit target for therapeutics against COVID-19, SARS-CoV-2 N protein has been widely reported to undergo phase separation *in vitro* in a salt-, pH- and RNA-dependent manner and *in cellular* (Cubuk et al., 2021; Iserman et al., 2020; Lu et al., 2021; Zhao et al., 2021) (Fig. 5C). Considering N and viral RNA forming higher-order structures that resemble the “beads on a string” pattern observed in infected cells (Klein et al., 2020), This behavior could potentially have *in vivo* implications, as it may facilitate the formation of ribonucleoprotein complexes (RNPs) in a manner reminiscent of the previously discussed replication compartments. What’s more, the SARS-CoV-2 N protein not only drives LLPS but also integrates as a client protein within the liquid phases formed by Ras-GTPase-activating protein SH3 domain-binding protein (G3BP). This interaction disrupts SG assembly, hinting at the N protein’s ability to recruit host SG components to bolster virus replication (Espinosa et al., 2020; Lu et al., 2021; Luo et al., 2021).

Primarily, the majority of instances where viral proteins drive LLPS have focused on the creation of membrane-less compartments, primarily for replication or assembly purposes. It is our expectation that in the near future, more cases of viral aggregation, which may not have initially been recognized as LLPS manifestations, will be reevaluated. Nonetheless, to instill confidence in LLPS as a potential therapeutic target, further in-depth investigations are warranted. These studies should delve into the intricate links between LLPS and various stages of viral life cycles, as well as the transition between phase-separated states such as liquid, gel, or glass-like structures that adapt to different infection events.

### Innate immune response

Innate immune signaling is initiated when cell surface or intracellular pattern recognition receptors identify exogenous pathogen-associated molecular patterns (PAMPs), triggering immune responses. Upon stimulation, these pattern recognition receptors recruit adaptors and effectors, forming higher-order assemblies. These assemblies, in turn, play a pivotal role in executing signal transduction and amplification. A prominent example is the cytosolic DNA sensor, cyclic GMP-AMP synthase (cGAS), which exhibits the tendency to form liquid-like droplets upon recognizing PAMPs. This phase separation phenomenon not only facilitates cGAMP production but also promotes innate immune signaling (Du and Chen, 2018; Liu et al., 2018). What’s even more intriguing is the observation that interferon-stimulated genes, including PKR, ADAR1, RNA-sensing RIG-I-like receptors (RIG-I, MDA5, LGP2), RNase L, and OAS, have been reported to colocalize with SGs during viral infections, as documented by various studies (Okonski and Samuel, 2013; Onomoto et al., 2012; Poblete-Durán et al., 2016). Given the strong correlation between SG formation and interferon production (Hu et al., 2010; Kim et al., 2019b; Manivannan et al., 2020; Onomoto et al., 2014), It is tempting to speculate that SGs, characterized by phase-separated RNA-binding proteins with low complexity sequence domains serving as the molecular foundation for their assembly (Molliex et al., 2015; Protter and Parker, 2016), may play a role in antiviral defense by serving as platforms for innate immune responses. Furthermore, recent studies have shown that SGs can extend their function beyond immune regulation by inhibiting viral replication (Paget et al., 2023). These findings highlight the adaptable role of SGs as cellular “buffers” which aid in maintaining cellular homeostasis by attenuating detrimental immune responses and viral replication (Paget et al., 2023). Consequently, phase separation emerges as an intricate adaptation mechanism that orchestrates dynamic responses, including the detection of foreign molecules and the activation or buffer of innate immune defenses during infections.

In response to host immune systems’ efforts to combat infections, pathogens have evolved a range of virulence strategies to evade host responses. One such strategy involves LLPS-mediated viral interference, often through the interaction of viral condensates with specific host proteins. The Epstein-Barr virus (EBV) provides an illustrative example of this. Within EBV, viral proteins EBNA2 and EBNALP are known to form liquid-like condensates at super-enhancer sites related to MYC and Runx3. In this context, they function as transcription factors, mediating both viral and host gene transcription (Peng et al., 2020b). Additionally, it’s important to note that certain viral proteins are known to form fibrillar aggregates, sequestering key host proteins. This sequestration serves various functions, such as (i) blocking host apoptosis and necroptosis pathways by forming alternative amyloid-based structures in conjunction with cellular RIP homotypic interaction motif-containing proteins (e.g., the M45 protein from murine cytomegalovirus, MCMV) (Pham et al., 2019); (ii) interfering with the assembly of canonical and non-canonical SGs (e.g., VP35 protein from the Ebola virus, EBOV) (Le Sage et al., 2017); and (iii) suppressing host RNA synthesis by blocking host transcription factors (e.g., nonstructural proteins from the Rift Valley fever virus, RVFV) (Le May et al., 2004).

### Bacterial dormancy and infection

Antimicrobial resistance is undeniably one of the most pressing challenges to human health in the coming decades. Bacterial infections, in many cases, have become increasingly difficult to treat, even when no genetic resistance is detected. These infections often tend to recur (Gollan et al., 2019). What’s even more concerning is the presence of persisting or dormant bacteria that can withstand drug treatments at concentrations that should be lethal, primarily by entering a state of antibiotic persistence (Fisher et al., 2017; Gollan et al., 2019). In essence, when antibiotics are administered, they effectively eliminate the actively dividing bacteria while sparing the dormant ones. A growing body of evidence indicates that dormant bacteria gain a phase transition, transforming their cytoplasm from a liquid-like state to a solid-like one, exhibiting properties of glass-forming liquids (Azaldegui et al., 2020; Munder et al., 2016; Parry et al., 2014). Significantly, this reversible solidification of the cytoplasm appears to be a critical survival strategy for cells under stress conditions (Rabouille and Alberti, 2017). Consequently, efforts to reverse the phase transitions in dormant bacteria hold great promise for innovative therapeutic interventions.

### Therapeutics: how to combat GoPS and LoPS-related diseases

As previously mentioned, the exploration of LLPS has garnered significant attention, partly because it implicates various proteins in the formation of condensates, which have been associated with a range of diseases, notably neurodegenerative disorders, cancer, and infectious diseases. While the precise cause-and-effect relationships between abnormal phase separation and these maladies remain incompletely understood, the conceptual framework of LLPS presents new prospects and promising therapeutic targets.Moreover, an increasing body of evidence seeks to unravel the precise links between the gain and loss of phase separation and disease pathogenesis. By categorizing aberrant phase separation into GoPS and LoPS, we aim to provide a more straightforward means of elucidating these connections. This approach not only simplifies the understanding but also adds a layer of intricacy to this captivating field of research, potentially revealing novel strategies for intervention and treatment.

In the current landscape of therapeutic approaches to tackle diseases associated with phase separation, two primary strategies have emerged, which target the “controllers” and “drivers” of LLPS, respectively (Brocca et al., 2020; Wheeler, 2020). The first category, the controllers, primarily include structural proteins like enzymes, cell surface receptors, nuclear hormone receptors, ion channels, and transporters. These proteins are subject to regulation via signaling pathways and various PTMs. In contrast, the phase-separation drivers encompass specific intrinsically disordered proteins (IDPs) and nucleic acids (DNA or RNA). Their interactions, whether it’s protein-protein or protein-nucleic acid, are indispensable for initiating LLPS. These therapeutic strategies provide new hope for addressing diseases linked to abnormal phase separation.

Traditionally, drug design and characterization have heavily relied on the structures of the underlying protein targets (Rask-Andersen et al., 2014). The analysis of the druggable proteome has shown a high level of structural coverage, with over 94% of targets having resolved structures (Hu et al., 2016; Lounnas et al., 2013; Rask-Andersen et al., 2014). In the context of phase separation, the controllers typically adopt a “scaffold-client” mode for stoichiometry regulation. Subject to various chemical modifications, scaffold proteins are prone to exhibit altered physicochemical properties, in which LLPS disappears with the condensate being dissolved or vice versa. Accumulating examples are known: (i) impaired LLPS exampled by phosphomimetic mutation of TDP-43 with decreased RNA splicing activity (LoPS) (Wang et al., 2018a), and serine phosphorylation and arginine methylation of FUS with reduced aggregation-prone character (LoPS) (Hofweber et al., 2018; Monahan et al., 2017); (ii) promoted LLPS exampled by phosphorylation of FMRP and CAPRIN1 as the switch for their co-phase separation and a determinant of sub-compartmentalization with RNA (GoPS) (Kim et al., 2019c), and Tau with enhanced phase transition (GoPS) (Wegmann et al., 2018).

As our understanding of aberrant modifications within phase-separated scaffolds linked to diseases expands, there is potential to identify the enzymes responsible for these modifications, such as kinases, phosphatases, methyltransferases, and demethylases, as viable therapeutic targets. On the other hand, client proteins can partition into condensates through interactions with the scaffolds, which can be quantified with a partition coefficient (Banani et al., 2016). This suggests the possibility of repurposing conventional drug targets to modulate the partitioning of client proteins into condensates. However, it remains a challenge to identify the relevant PTM enzymes and specific small molecule inhibitors within a simple, sensitive, and effective system. The evaluation of the effects of PTM interventions often necessitates multiple tests in disease models. Moreover, obtaining the correct composition within the condensates is essential for understanding their biological functions.Recent evidence highlights the role of SH2-containing protein tyrosine phosphatase-2 (SHP2) as a prime example, demonstrating how phase separation serves as a gain-of-function mechanism in the pathogenesis of SHP2-associated human diseases. This research also opens the door to the possibility of therapeutically targeting phase separation using small molecule drugs like SHP2 allosteric inhibitors (Zhu et al., 2020).

**Figure 6.**
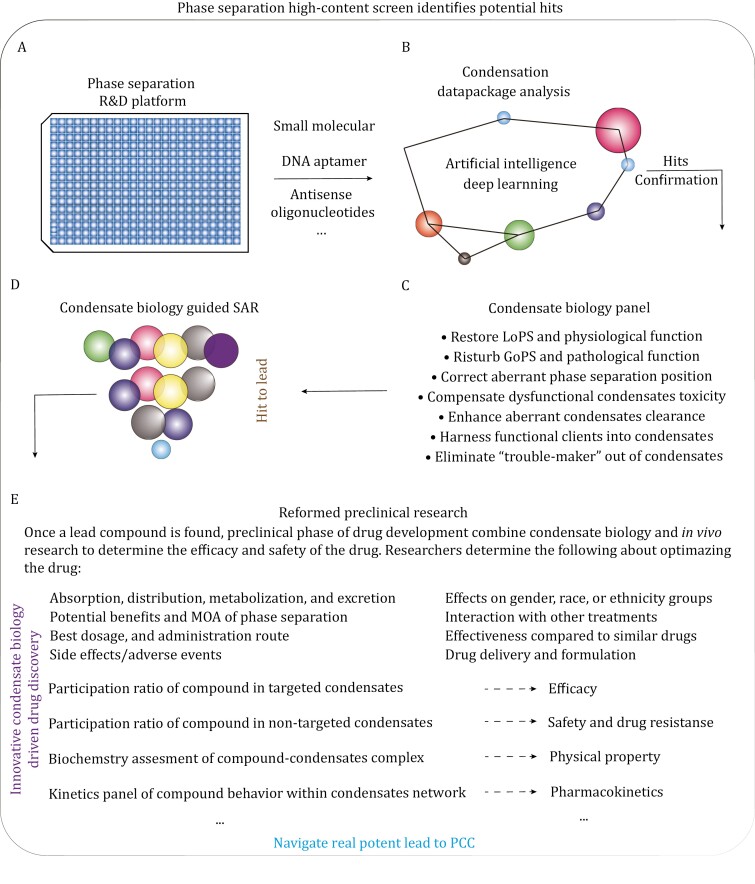
Potential therapeutic intervention targeting aberrant phase separation via high-content screening. Utilizing cell-based or/and protein-based high-content screening, it is conceivable to identify small molecular compounds directly targeting aberrant phase separation (LoPS/GoPS) via physical interactions with components within condensates *in vitro* or phenotypic screening for effects on condensates *in cellular*. Hit compounds can be further evaluated as any lead compounds that are towards clinical utilization. (PCC, preclinical candidate; SAR, structure–activity relationships; R&D, research and development).

However, IDPs associated with LLPS represent attractive but challenging drug targets. Despite their abundance in the human proteome and their apparent involvement in disease pathogenesis, IDPs pose a dilemma for researchers aiming to identify potential targets for conventional drug design. Some exceptions, such as histone deacetylases, nuclear receptors, and transcription factors with relatively high disorder content, are known to be druggable targets (Hu et al., 2016; Metallo, 2010; Uversky, 2012). Nevertheless, the efforts to target these canonically undruggable IDPs tend to focus on finding binding sites within non-enzymatic structured domains (Makley and Gestwicki, 2013), cryptic binding sites (Vajda et al., 2018), or even transient binding sites (Chong et al., 2018). Fortunately, LLPS introduces a potential new concept for traditionally undruggable IDPs through a physicochemical mechanism. This approach involves identifying drugs that directly target aberrant phase separation (LoPS/GoPS) without requiring specific binding pockets (Babinchak et al., 2020; Kim et al., 2019a; Shen et al., 2021; Wheeler et al., 2019). Utilizing cell-based or/and protein-based high-content screening, it is speculative to identify a class of small compounds directly targeting aberrant phase separation via partition to condensates *in vitro* or phenotypic screening for effects on condensates *in cellular*, further applied feasibly to clinical tests (Fig. 6). With the establishment of visual models combined with high-content imaging technology *in vitro* as well as *in cellular*, the large-scale screening of phase-separation modulators is becoming more convenient and widely used. Among them, cell-based drug screening is exemplified by the system targeting phase separation of FUS-associated defects, in which lipoamide and lipoic acid were identified to dramatically reduce the aggregation of SG proteins, perhaps recovering neuronal defects (Wheeler et al., 2019). Additionally, recent research provides a pharmacological strategy based on phase separation to target the undruggable steroid receptor coactivator-1 (SRC-1) and suppress the oncogenic transcription activity of Yes-associated protein (YAP), underscoring the potential of phase-separation-targeted therapeutics as a novel and potent approach for addressing traditionally undruggable targets (Zhu et al., 2021).

Remarkably, there is a distinct dearth of reported universal drug screening and development platforms designed to tackle abnormal phase separation in biological systems. In a recent study, the researchers honed in on the aberrant phase separation induced by fusion genes, revealing their acquisition of phase separation domains (PSs) and DNA-binding domains (DBDs), intricately linked to the tumorigenic process (Wang et al., 2023). In response to this challenge, the authors have ingeniously devised a versatile high-throughput drug screening platform, aptly named DropScan (Wang et al., 2023). Capitalizing on this platform, they conducted extensive screenings and multidimensional analyses of phase separation data, culminating in the identification of LY2835219 as a novel and highly specific compound for regulating the aberrant phase separation of PS-DBD fusion genes and modulating the expression of cancer-related genes (Wang et al., 2023). This study serves a dual purpose by shedding light on the significance of PS-DBD fusion genes in the genesis of cancer, and more prominently, by introducing a versatile and groundbreaking drug screening platform. This innovative platform can be customized to suit the distinct characteristics of individual aberrant phase separation disease targets. It seamlessly incorporates modules for recognizing phase separation and applying deep learning techniques, expediting data analysis. Notably, it is poised to address the challenges presented by complex targets such as neurodegenerative diseases, transcription factors, nuclear receptors, and other targets requiring innovative and physiologically relevant drug screening platforms.

A noteworthy advantage of DropScan lies in its ability to harness the unique pathological profiles and status of the target molecules themselves, accurately identifying drug candidates that conventional methods may inadvertently overlook. This competitive edge has the potential to significantly accelerate traditional drug development processes and potentially reshape the landscape of addressing complex drug targets. Furthermore, the platform showcases impressive versatility in its applications. Through the modification and reconfiguration of its modules, it can seamlessly pivot towards the development and screening of various drug types, including PROTAC, molecular glue, nucleic acid-based drug, and more. In summation, DropScan presents a multifaceted array of benefits and novel insights, encapsulated in its universality, scalability, adaptability, throughput, and precision, making a substantial contribution to the arena of drug discovery and development.

On the flip side, targeting condensates with small molecules poses its own set of challenges, extending beyond concerns related to druggability. Specificity is a crucial issue that must be addressed. It is not yet clear whether a small molecule designed for a specific target condensate would exclusively interact with that condensate and not affect other unrelated condensates. There is a precedent for this concern, as 1,6-hexanediol (1,6-HD) has been shown to impact various condensates, raising questions about the selectivity of small molecules in this context (Düster et al., 2021; Itoh et al., 2021; Wheeler et al., 2016). Accumulating knowledge of coexisting immiscible condensates, exemplified by the nucleolar sub-compartments (Feric et al., 2016), perhaps provides a clue for allowing specific partition of a small molecule into clients via different physicochemical properties of scaffolds (Wheeler and Hyman, 2018).

Condensates, once a relatively overlooked biological phenomenon, have now gained significant attention in the pharmaceutical industry. Leading the way is Dewpoint Therapeutics, along with several startups like Nereid Therapeutics, Transition Bio, Eternbio, Medusa Therapeutics, Nuage Therapeutics, Granule Therapeutics and NuPhase Therapeutics. These companies are actively working to manipulate membraneless condensates associated with disease pathogenesis. Established pharmaceutical giants like Roche, Pfizer, Merck, Bayer, Insilito Medicine, and Novo Nordisk are also collaborating with these startups or academics to explore the potential of condensates in drug discovery and development. Dewpoint Therapeutics, rooted in the life sciences, focuses on dissolving disease-associated condensates or directly blocking their formation using high-throughput screening of small molecules and *in vitro* pharmacology. On the other hand, Nereid Therapeutics, with a physics-centric approach, invests in developing a range of tools, including optogenetic platforms and biomimetic systems, to understand the physicochemical mechanisms governing the formation of liquid-like assemblies and material state transitions. However, it’s essential to acknowledge that manipulating transient assemblies or their composition for therapeutic purposes may require a more in-depth understanding of condensate biology. We are still far from a complete understanding of the complex biology of membraneless organelles. Condensate biology not only emerges as a novel field but also prompts a rethinking of cell biology, shedding new light on these intriguing structures.

## Conclusion and outlook

In summary, we have systematically accumulated a robust framework, comprehensive insights, and compelling potential applications through our primary exploration of this field. The rapid advancements in genetics and other areas of biology have enabled the identification of genetic causes of diseases at the intricate base-pair scale. However, many of these discoveries have not provided specific insights into the relevance or causality of these genetic factors, let alone potential therapeutic interventions. The intricate web of cellular functional networks challenges us to continually delve into the intricate workings of each molecular unit.

In this review, we have diligently endeavored to provide a comprehensive summary of the recent advancements in the correlation between aberrant phase separation and diseases, as well as the exploration of potential therapeutic strategies. The prevalence of biomolecular condensate phenomena underscores their pivotal role in cellular function. Over the past decade, researchers in this field have harnessed a wide array of experimental techniques, both within and outside cellular contexts, integrating knowledge from diverse disciplines to advance our comprehension of biological phase separation. Looking forward, the field is actively exploring avenues to foster further progress and embark on a new phase of discovery and innovation. In this review, we highlight the emerging developments in connecting aberrant phase separation with diseases and the potential therapeutics that may stem from this understanding. The ubiquity of condensation has robustly demonstrated their significance in cellular function. Over the past decade, researchers in this field have leveraged an array of experimental techniques within and outside cellular environments, integrating knowledge across diverse disciplines to advance our understanding of biological phase separation (Seydoux et al., 2023). With the growing interest of pharmaceutical industries in addressing disease through the lens of phase separation, there is much promise in combating diseases covering cancer, neurodegenerative disease, and infectious diseases. Emerging methods and strategies have been developed to accelerate early drug discovery by manipulation and modification of phase separation (Li et al., 2022; Wang et al., 2023; Xu et al., 2021). Several leading pharmaceutical companies are already actively exploring this area. They are investing in research and development to identify small molecules or modulators that specifically target phase separation components involved in disease processes. These efforts aim to pave the way for innovative therapeutics that can precisely control phase separation in cellular processes.

The vision for the future of condensate research in the medical and pharmaceutical fields encompasses the development of personalized medicine approaches. By understanding the specific phase separation patterns in individual patients, tailored treatments can be designed to address their unique disease mechanisms. This personalized approach holds immense potential for improved patient outcomes and more precise, effective medications.

In conclusion, the future of research in phase separation within the medical and pharmaceutical realms is highly promising. The ongoing advancements in this field open the door to transformative therapies and groundbreaking medications, potentially reshaping our approach to disease treatment and patient care. In the forthcoming years or even decades, our optimism lies in unraveling the intricate code of diseases and the anomalies in phase separation. As we achieve this profound understanding, it may pave the way for the development of effective cures for a multitude of ailments, offering hope for improved healthcare and the betterment of human well-being.

## Data Availability

Not applicable.

## Abbreviations

1,6-HD: 1,6-hexanediol
AD: Alzheimer’s disease
ALP: autophagy-lysosome pathway
ALS: amyotrophic lateral sclerosis
AR: arogen receptor
α-Syn: alpha-Synuclein
cGAS: cyclic GMP-AMP synthase
CAM: chaperon-mediated autophagy
DAXX: death-domain-associated protein
EBOV: Ebola virus
EBV: Epstein-Barr virus
ENL: eleven-nineteen-leukaemia protein
ER: endoplasmic reticulum
FA: Friedreich’s ataxia
FTD: , frontotemporal dementia
FUS: fused in sarcoma
G3BP: Ras-GTPase-activating protein SH3 domain-binding protein
GoPS: gain of phase separation
HIV-1: human immunodeficiency virus 1
HSV-1: herpes simplex virus 1
HD: Huntington’s disease
HTT: Hutingtin
IAV: Influenza A virus
IBs: inclusion bodies
IDR: intrinsic disordered region
IDPs: intrinsically disordered proteins
ISGs: interferon-stimulated genes
LLPS: liquid-liquid phase separation
LoPS: loss of phase separation
MCMV: murine cytomegalovirus
mHTT: mutant Hutingtin
NAC: non-amyloid-β component
NBs: Negri bodies
NC: nucleocapsid protein
PAMPs: pathogen-associated molecular patterns
PCC: preclinical candidate
PD: Parkinson’s disease
polyQ: poly-glutamine
PTM: post-translational modification
RABV: rabies virus
R&D: research and development
RCs: replication compartments
RVFV: Rift Valley fever virus
RHIM: RIP homotypic interaction motif
RSV: respiratory syncytial virus
SAR: structure-activity relationships
SG: stress granule
SHP2: SH2 containing protein tyrosine phosphatase-2
SPOP: speckle-type POZ protein
SRC-1: steroid receptor coactivator-1
TDP-43: TAR DNA binding protein 43
UPS: ubiquitin-proteasome system
VSV: vesicular stomatitis virus
YAP: Yes-associated protein.
